# Laparoscopic distal pancreatectomy in a patient with aberrant splenic artery originating from the superior mesenteric artery: A case report

**DOI:** 10.1097/MD.0000000000025704

**Published:** 2021-05-07

**Authors:** Hiroyuki Ishida, Yoshiya Ishikawa, Keiichi Akahoshi, Hiroki Ueda, Koichiro Morimoto, Hironari Yamashita, Kosuke Ogawa, Hiroaki Ono, Atsushi Kudo, Shinji Tanaka, Minoru Tanabe

**Affiliations:** a Department of Hepatobiliary and Pancreatic Surgery; b Department of Molecular Oncology, Tokyo Medical and Dental University, Tokyo, Japan.

**Keywords:** anatomical variants, case report, dorsal pancreatic artery, laparoscopic distal pancreatectomy, splenic artery

## Abstract

**Rationale:**

Splenic artery originating from the superior mesenteric artery is extremely rare. Because of this, its significance in laparoscopic distal pancreatectomy has never been reported. Here, we present the first case of laparoscopic distal pancreatectomy in a patient with a splenic artery arising from the superior mesenteric artery.

**Patient concerns:**

A 46-year-old Japanese woman with type 2 diabetes mellitus presented with worsening glycemic control. Abdominal ultrasonography revealed a pancreatic tail mass.

**Diagnoses:**

The patient was diagnosed with pancreatic neuroendocrine tumor by endoscopic ultrasound-guided fine needle aspiration. Preoperative computed tomography showed that the splenic artery with branches of dorsal pancreatic artery originated from the superior mesenteric artery.

**Interventions:**

The patient underwent laparoscopic distal pancreatectomy. Prior to pancreatectomy, the splenic artery and its dorsal pancreatic branches were clamped using the superior and inferior approaches, respectively, to avoid bleeding and congestion.

**Outcomes:**

The postoperative course was uneventful.

**Lessons:**

Preoperative evaluation of anatomical variants and development of strategies are important to avoid intraoperative complications in pancreatic surgery. Our results revealed that laparoscopic distal pancreatectomy can be performed safely by strategic approach even in a patient with a rare aberrant splenic artery.

## 1 Introduction

The splenic artery (SpA) is a large and relatively constant branch of the celiac trunk. Although rare, anatomical variations of the SpA, such as an SpA originating from the superior mesenteric artery (SMA), still exist. Previous reports have shown that the incidence of this SpA variation was only between 0.03% and 1.2%.^[1–4]^ Nevertheless, preoperative planning in patients with aberrant SpA is important as anatomical variations could lead to intraoperative vascular injury and prolonged operation time. However, to the best of our knowledge, the clinical significance of the aberrant SpA arising from the SMA in laparoscopic distal pancreatectomy (LDP) has not been documented. Here, we present the first case of LDP in a patient with this anatomical variation.

## 2 Case presentation

**Figure 1:**
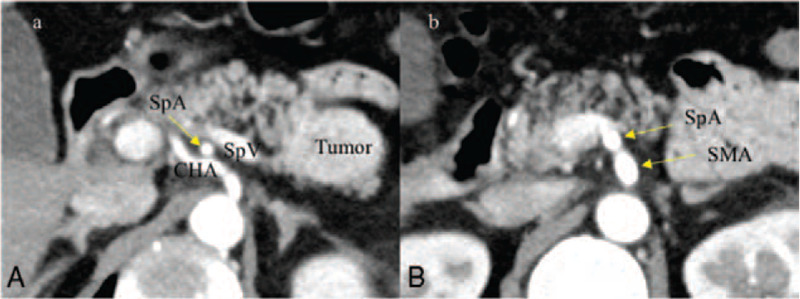
Preoperative CT images. A: A high enhanced tumor measuring 45 mm in a diameter at the pancreatic tail was detected. SpA was running near the root of CHA. B: SpA originating from SMA was running dorsally to the pancreas. *CHA* = common hepatic artery, *SpA* = splenic artery, *SpV* = splenic vein.

**Figure 2:**
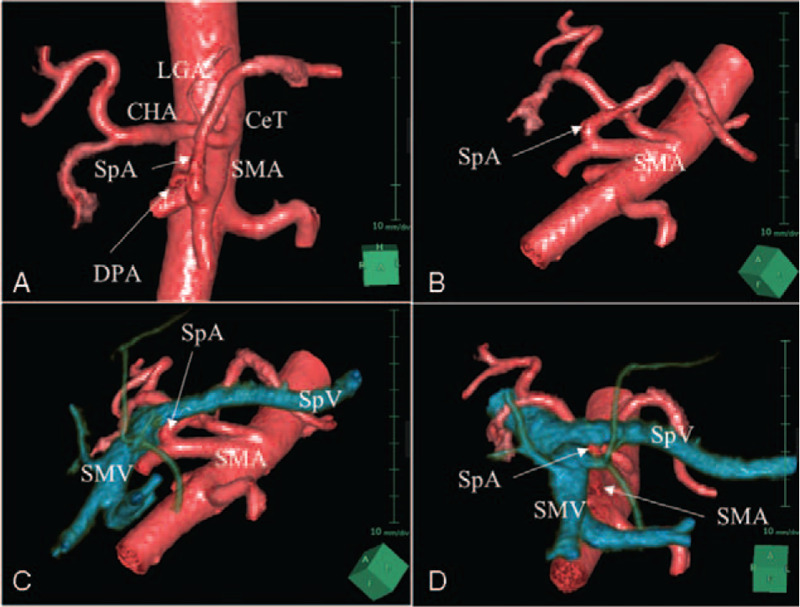
Preoperative 3D-CT images. A: CeT had only two branches: LGA and CHA. SpA was originating from the SMA with DPA from its origin. B: The anatomical relationship between SpA and SMA was revealed in a left anterolateral view. C: SpA was originating at the level of the inferior border of SpV and ascending dorsally to SpV. D: The origin of SpA was confirmed from the inferior border of the pancreas. *CeT* = celiac trunk, *CHA* = common hepatic artery, *DPA* = dorsal pancreatic artery*, LGA* = left gastric artery*, SpA* = splenic artery, *SpV* = splenic vein.

A 46-year-old Japanese woman with type 2 diabetes mellitus presented with worsening glycemic control. Hemoglobin A1c level was elevated at 8.2%, confirming the poor glycemic control. The patient had no previous history of abdominal surgery. Abdominal ultrasonography revealed a pancreatic tail mass. Histopathologic analysis of the mass taken through endoscopic ultrasound-guided fine needle aspiration was performed, and the patient was diagnosed with pancreatic neuroendocrine tumor. Preoperative computed tomography (CT) showed a 45-mm enhanced tumor at the pancreatic tail with an SpA originating from the SMA at the level of the inferior border of the splenic vein (SpV) and ascending along the SMA toward the superior pancreatic border dorsally (Fig. 1). The SpA had several branches of the dorsal pancreatic artery (DPA) extending into the pancreatic body. The celiac trunk had only two branches: the left gastric artery and the common hepatic artery (CHA). Three-dimensional (3D) CT images were constructed and visualized using the SYNAPSE VINCENT image processing software (FUJIFILM Medical Co., Tokyo, Japan) (Fig. 2). The anatomical relationship between SpA and SpV was revealed more clearly in a different view by using 3D-CT images.

**Figure 3:**
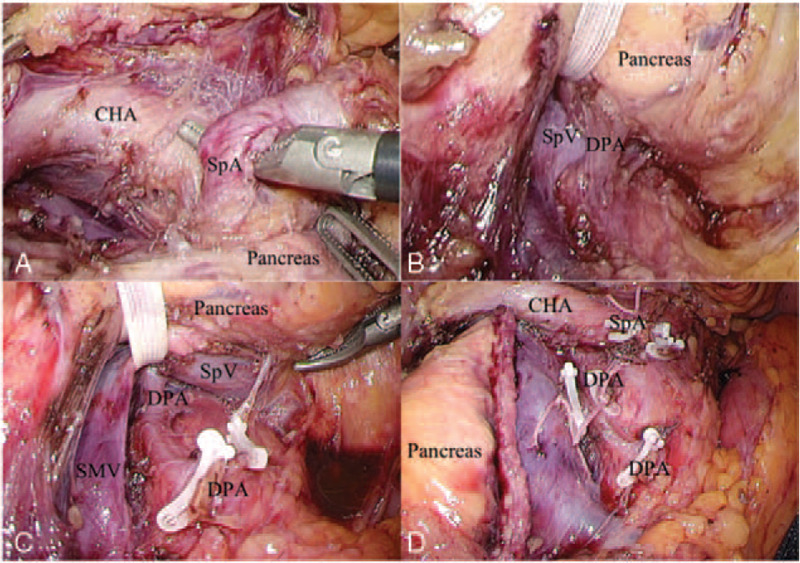
Intraoperative findings. A: SpA was identified near the root of CHA at the superior border of the pancreas by superior approach. B: One branch of DPA was identified at the caudal side of SpV by inferior approach. C: Another branch of DPA was found when SpV and pancreatic body were lifted ventrally. D: The stumps of SpA and two branches of DPA were revealed after pancreatic transection and SpV division. CHA = common hepatic artery, DPA = dorsal pancreatic artery, SMV = superior mesenteric vein, SpA = splenic artery, SpV = splenic vein.

We performed LDP for this case. Prior to transection of the pancreas, the SpA was first clamped from the superior border of the pancreas. To do this, the greater omentum was opened and the stomach was pulled up to the abdominal wall by the round ligament and a Penrose drain, after obtaining pneumoperitoneum. By dissecting the peritoneum along the superior pancreatic border, we identified the CHA. Dissection was continued towards the root of the CHA, where the SpA was recognized near the CHA. By dissecting the nerve fibers between the CHA and SpA, we successfully approached the SpA at the superior border of the pancreas to allow clamping (Fig. 3).

Consequently, the DPA from the origin of the SpA was then clamped from the inferior border of the pancreas. To do this, the superior mesenteric vein was exposed below the pancreatic neck. After identifying the SpV, one branch of the DPA running on the caudal side of the SpV was resected. The pancreatic body and the SpV were then lifted ventrally by taping at the level of the SMA. Under a magnified laparoscopic caudo-dorsal view, the DPA originating from the SpA at the cranial side of the SpV was identified and clamped. We did not dissect the SMA nerve plexus to identify the origin of the SpA because the resection of SpA at the level of its origin was not mandatory in this case.

The arterial blockade was completed by both superior and inferior approaches. After the transection of the pancreas with a linear stapler and the division of the SpV at its confluence, the DPA and SpA were confirmed more precisely and resected. The distal pancreas was subsequently dissected from the retroperitoneum and the specimen was retrieved. The operation time was 410 minute, with a blood loss of 150 mL. The postoperative course was uneventful.

## 3 Discussion

This is the first report of LDP for a patient with the aberrant SpA originating from SMA. Although the minimally invasive approach has been reported previously in an aneurysm originating from the aberrant SpA,^[5]^ there have been no studies regarding its use for LDP on this variant.

We have previously reported that the anatomical relationship between the SpA and the pancreatic parenchyma influences LDP, sometimes leading to increased difficulty in performing the procedure.^[6]^ It was suggested that when the pancreatic parenchyma is ventral to the bifurcation of the SpA and CHA (i.e., the SpA origin was buried dorsal to the pancreas), SpA may be relatively difficult to identify. Several studies have recommended that an inferior approach may be used in performing LDP for such cases.^[7,8]^ In our case, the DPA arising from the origin of the SpA was also identified via the inferior approach.

In open surgery, exposure of the aberrant origin of SpA by the inferior approach was cumbersome. When Fiorello et al. performed a surgery for aberrant SpA by open distal pancreatectomy, transection of the pancreas and division of the SpV was crucial to better visualize the aberrant SpA,^[3]^ thus leading to increased risk of bleeding and pancreatic congestion after the SpV division. In contrast, a laparoscopic caudo-dorsal view could offer the advantage of approaching the SpA and DPA by the inferior approach, which is easier, while minimizing risk of complications.

In our case, the SpA originated at the level of the inferior border of the SpV but ran near the root of the CHA. In addition, the DPA arose from the aberrant SpA at the dorsal side of the SpV with several branches to the pancreatic body. Therefore, arterial blockade was done via superior and inferior approaches for the SpA by exposing the CHA to its root side and then DPA, respectively. Our results demonstrate that this combination strategy is useful during LDP, even in patients with aberrant SpA.

In conclusion, using a combined superior and inferior approach, LDP can be performed safely for a patient with aberrant SpA originating from the SMA.

## Acknowledgments

We would like to thank Editage (www.editage.com) for English language editing.

## Author contributions

**Conceptualization:** Hiroyuki Ishida, Yoshiya Ishikawa.

**Writing – original draft:** Hiroyuki Ishida, Yoshiya Ishikawa, Keiichi Akahoshi.

**Writing – review & editing:** Hiroki Ueda, Koichiro Morimoto, Hironari Yamashita, Kosuke Ogawa, Hiroaki Ono, Atsushi Kudo, Shinji Tanaka, Minoru Tanabe.
